# Addressing Osteonecrosis: A preventable catastrophe - A multi-center study

**DOI:** 10.12669/pjms.39.1.5985

**Published:** 2023

**Authors:** Mafaza Alam, Zahid Mehmood Khan, Laima Alam, Varqa Faraid, Adnan Khan

**Affiliations:** 1 Mafaza Alam, Registrar Operative Dentistry and Endodontics, Armed Forces Institute of Dentistry (AFID), Rawalpindi, Pakistan; 2 Zahid Mehmood Khan, FCPS Operative Dentistry and Endodontics, Armed Forces Institute of Dentistry (AFID), Rawalpindi, Pakistan; 3 Laima Alam, FCPS Gastroenterology, MRCP (UK), Bahria Town international Hospital, Rawalpindi - Pakistan; 4 Varqa Faraid, Demonstrator Oral Medicine, School of Dentistry, Islamabad - Pakistan; 5 Adnan Khan, Registrar Maxillofacial Surgery, Armed Forces Institute of Dentistry (AFID), Rawalpindi, Pakistan

**Keywords:** Chemotherapy, Bisphosphonate-related osteonecrosis of the jaw, Osteonecrosis, Radiotherapy

## Abstract

**Objectives:**

To assess the knowledge of medical doctors about osteonecrosis who prescribe radiotherapy and bisphosphonates and dentists who receive these patients with such risk factors.

**Methods:**

This cross sectional multicenter study was carried out from 15^th^ October 2021 to 20^th^ November 2021 in different set-ups of Pakistan. A validated and piloted questionnaire was sent to dental and non-dental doctors working in different set-ups of Pakistan through email. All data was analyzed in SPSS version 22 with p value <0.05 being significant.

**Results:**

A total of 400 completed responses were received. Only 58% and 67% of the participants were actively educating their patients regarding the hazards of bisphosphonate and radiotherapy, respectively whereas only 45% of the medical doctors referred their patients to dentists before prescribing bisphosphonates and/or radiotherapy. Although the medical doctors had a statistically better knowledge of the definition of osteonecrosis, overall both the dental and non-dental doctors performed poorly regarding answering the questions pertaining to definition, clinical features and risk factors. The dental doctors also showed a poor performance for more technical and in depth questions that was statistically related to lesser work experience, working in a tertiary care facility and previous exposure to such patients.

**Conclusion:**

The inadequate awareness of dentists and physicians about the prevention and management of osteonecrosis of jaw is alarming. Efforts should be undertaken to raise the knowledge of dentists and physicians in this regard.

## INTRODUCTION

The impeding blood flow to the jaw bones lead by radiation therapy or drugs that disrupt bone turn over due to their anti-responsive functions like bisphosphonates are the classical causes of osteonecrosis of jaw. A dramatic exposure of necrotic bone after dental extractions distressed dentists as well as physicians in patients undergoing radiotherapy, chemotherapy, high doses of steroids as well as bisphosphonates.^1^,^2^

The early cases of Medication Related Osteonecrosis of Jaw (MRONJ) were reported in 2003 by dental surgeons.^3^,^4^ Along with bisphosphonates, its occurrence is associated with other drugs classes like Anti Resorptive Drugs (Denusumab), Anti Angiogenic Drugs (Sunitinib and Sorafenib), Human Monocolonal Antibodies (Bevacizumab) And Anti-VEGF Agents.^5^ The incidence of Bisphosphonate Related Osteonecrosis of Jaw (BRONJ) has been reported to be 0.01 to 6.7% and is expected to grow continuously with increase in the prescription of bisphosphonates for various diseases like osteoporosis, multiple myeloma, osteogenesis imperfect, osteitis deformans, bone metastasis and Paget’s disease.^5^

**Fig.1:**
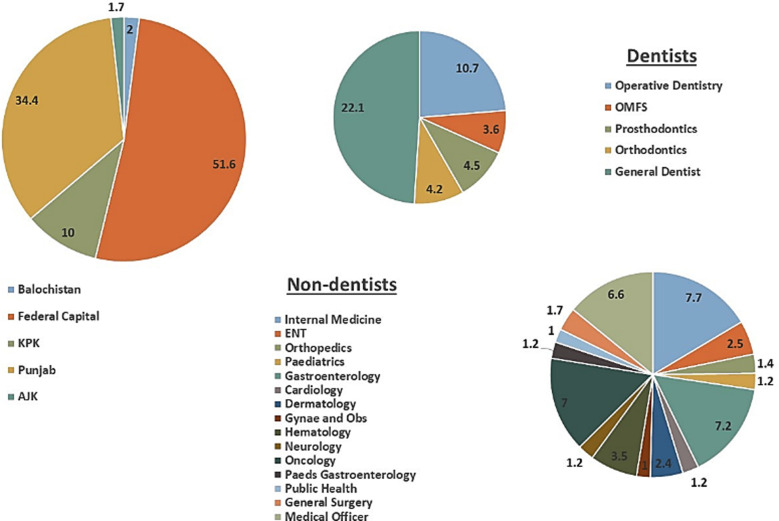
Distribution of specialties and location of the participants.

Radiation therapy deteriorates the DNA of swiftly proliferating cancerous as well as healthy cells non-selectively. Tormented bone healing causing osteo-radio necrosis can be caused by this lack of customary activity of bone cells as well. The areas exposed to radiation therapy encounter reduced perfusion leading to persistent non healing wounds.^6^

Due to the ongoing pandemic and huge spike in the use of corticosteroids and consequently bisphosphonates, the risk for developing osteonecrosis has largely increased.^7^ Osteonecrosis has a poor prognosis with a long term therapy but it is completely preventable if timely referrals, correct treatment planning and prompt decisions are made by the health care providers.^8^ Guidelines for staging and management of patients with bisphosphonates therapy have been published by *American Association of Oral and Maxillofacial Surgeons* (AAOMS) to educate the clinicians about the management of such patients and clinical presentation of BRONJ.^4^ Regardless of the available guidelines, several studies show an alarming deficient awareness of dentists and other medical doctors regarding this nerve wracking disease.^8^

This study is aimed to assess the knowledge of medical doctors about osteonecrosis who prescribe radiotherapy and bisphosphonates and dentists who receive these patients with such risk factors. No such study has been carried out in Pakistan which makes this a highly innovative topic owing to the current health scenario.

## METHODS

This cross sectional multi-center study was carried out by enrolling dental and non-dental doctors working in different set-ups of Pakistan through convenience sampling after acquiring ethical approval from the concerned department (IRB 90/Trg-ABP1K2 dated 14-10-2021). The survey was run from 15^th^ October 2021 to 20^th^ November 2021 by sending a validated and piloted questionnaire through email to the participants currently working in Combined Military Hospital Rawalpindi, Bahria International Hospital Rawalpindi, Pakistan Institute of Medical Sciences Islamabad and School of Dentistry Islamabad. Doctors from basic medical sciences and incomplete surveys were excluded.

The questionnaire was developed by MA and ZM after a detailed study of the available literature.^8^-^10^ It was scrutinized by two medical education experts for content validity and piloted on ten subjects for test purposes.^9^,^10^ The questionnaire consisted of three parts; basic demographics, experience with osteonecrosis and questions regarding basic knowledge that applied to both dental and non-dental doctors, and technical questions for testing the knowledge of the dental participants.

The sample size was calculated by OpenEpi sample size calculator with a 5% margin of error, 95% confidence interval and a 50% frequency of outcome factor. Quantitative data was expressed as frequency and percentages and comparisons were done using chi square statistics. All data was analysed in SPSS version 19 with p value <0.05 being significant.

## RESULTS

A total of 400 completed performas were received out of 700 sent, making a response rate of 43%. The participants were divided in to two equal groups of dentists and medical doctors, out of which 156 (39%) were males and 244 (61%) were females (Table-I). Majority of the participants were working in tertiary care facility (66.3%) with 1-4-year experience (54.4%). Less than half of the practicing doctors were referring patients for a dental checkup before initiating radiotherapy or bisphosphonate. About 58% and 67% of the doctors were actively educating their patients regarding radiation and bisphosphonate related osteonecrosis, respectively, although the reported rate of osteonecrosis for this study was as high as 37%.

**Table-I:** Basic demographics of the participants enrolled

Variables		Frequency (n)	Percentage (%)
Gender	Male	156	39
	Female	244	61
Specialty	Dentists	200	50
	Non-dentists	200	50
Experience	1-4 years	218	54.4
	5-10 years	142	35.4
	>10 years	40	10
Set-up	BHU	42	10.5
	District Health Unit	28	7
	Tertiary Care Hospital	266	66.3
	Private Clinic	64	16
No. of patients on bisphosphonates	None	170	42.4
	1-10	164	41
	>10	66	16.5
Ever heard the term radiation related osteonecrosis	Yes	297	74
	No	103	26
Ever heard the term bisphosphonates related osteonecrosis	Yes	317	79
	No	83	21
	No information	62	15.5
	Books	162	40.4
	Seminars	17	4.2
	Experience	2	0.5
	Academic journals	151	37.7
	Peers	6	1.5
Source of information regarding radiation related osteonecrosis	No information	94	23.4
	Books	151	37.7
	Seminars	18	4.5
	Experience	2	0.5
	Academic journals	128	32
	Peers	7	1.7
Do you refer patients to a dentist before bisphosphonate therapy (Do you receive such patients if Dentist)?	Yes	179	45
	No	221	55
Do you refer patients to a dentist before radiation therapy (Do you receive such patients if Dentist)	Yes	175	44
	No	225	56
Do you inform patients about osteonecrosis before starting them on bisphosphonates	Yes	268	67
	No	132	33
Do you inform patients about osteonecrosis before starting them on radiation	Yes	234	58.4
	No	166	41.6
Ever received patient with bisphosphonate related osteonecrosis?	Yes	149	37
	No	251	63
Ever received patient with radiation related osteonecrosis?	Yes	106	26.6
	No	294	73.4
Ever made a referral or received osteonecrosis?	Yes	133	33
	No	267	67
Do you take bisphosphonates history at dental appointment? (For dentists only)	Yes	138	69
	No	62	31

Although the medical doctors had a statistically better knowledge of the definition of osteonecrosis, overall both the dental and non-dental doctors performed poorly regarding answering the questions pertaining to definition, clinical features and risk factors (Table-II). The dental doctors also showed a poor performance for more technical and in depth questions that was statistically related to lesser work experience, working in a tertiary care facility with good patient output and previous exposure to patients with osteonecrosis of the jaw (Table-III).

**Table-II:** Performance of the participants regarding knowledge of Osteonecrosis of the jaw.

Vignette	No. of correct answers (n=400)		P value (dentists vs non-dentists)

	Dentists	Non-dentists	
Definition of osteonecrosis	45(11.3)	145(36)	<0.001
Clinical features	81(20)	30(7.5)	<0.001
Risk factors	96(24)	53(13.3)	<0.001

***Questions pertaining to dentists only (n=200)***			

*Vignette*	*No. of correct answers*	*No. of incorrect answers*	*P value (correct vs incorrect)*

Guidelines for surgical treatment	87(43.5)	112(56)	<0.001
Guidelines for endodontic treatment	83(41.5)	117(58.5)	<0.001
Suitability of invasive dental treatment with Intravenous bisphosphonate therapy	157(78.5)	43(21.5)	<0.001
Suitability of invasive dental treatment with oral bisphosphonate therapy for >4 years	108(54)	92(46)	0.09
Suitability of invasive dental treatment with oral bisphosphonate therapy for <4 years	112(56)	88(44)	0.26

**Table-III:** Relation of different variables with correct answers among participants with dental background.

Areas of tested knowledge	Experience	Set up	Previous experience with bisphosphonates
Risk factors	0.86	0.47	<0.001
Clinical features	<0.001	0.23	0.07
Definition	0.53	<0.001	<0.001
Surgical management guidelines	0.004	<0.001	<0.001
Endodontic management guidelines	0.003	<0.001	<0.001
Suitability of invasive dental treatment with oral bisphosphonate therapy for <4 years	0.014	<0.001	<0.001
Suitability of invasive dental treatment with oral bisphosphonate therapy for >4 years	0.89	<0.001	0.001
Suitability of invasive dental treatment with Intravenous bisphosphonate therapy	0.26	0.001	0.001

## DISCUSSION

The number of cases of osteonecrosis have increased due to the increased use of bisphosphonates, steroids and radiation therapy to head and neck.^3^,^11^ A high level of knowledge is required to prevent and manage the cases of osteonecrosis of jaw.^12^,^13^ After a thorough literature review, to our best knowledge, it is the first study of its kind to compare the knowledge of radiation and medication related osteonecrosis of jaw of dentists and physicians in Pakistan.

Over all the study disclosed an alarming level of lack of knowledge among physicians and dentists in regards to this disease. Our study revealed that 36% of non-dental doctors (including specialists and trainees from the departments of internal medicine, ENT, Orthopedics, Gastroenterology, Cardiology, Dermatology, Oncology, Hematology, General surgery and Pediatrics) knew correctly of the term osteonecrosis of the jaw, whereas, only 7.5% were aware of the clinical features of the disease. The risk factors were known to only 13.3% of the doctors with poor knowledge regardless of the experience, although 57.5% of the physicians had patients on bisphosphonates therapy. In a study done in Korea comprising of doctors from the departments of oncology, endocrinology, orthopedics, rheumatology and family medicine, only 22% had heard of the term bisphosphonates related osteonecrosis of jaw with no difference of level of awareness among different specialties. Whereas only 30% made dental referrals of patients before starting them up on bisphosphonate prescription.^9^ Likewise, in a study conducted in Saudi Arabia only 31.5% physicians were aware of the disease while more than 50% were already prescribing bisphosphonates.^10^

In a study conducted in Brazil, less than 10% of physicians could correlate the signs and symptoms of medicine related osteonecrosis of the jaw but physicians attending cancer patients had a better understanding of the disease.^5^ Similarly, a study conducted in Lebanon showed that 37.5% of the physicians were unaware of BRONJ whereas 63.5% were actively treating patients with bisphosphonates for malignances.^14^,^15^ Our study revealed that academic books followed by journals were the major source of information regarding BRONJ and radiation related osteonecrosis of jaw. Whereas according to the Korean study majority of the participants gained knowledge of the disease through journals followed by seminars.^9^

In our study only 11.5% dentists knew the correct definition of osteonecrosis. 20% of the dentists were aware of the clinical features with a dismal situation of poor knowledge with higher experience. 24% of the dentists were familiar with the risk factors of the disease which was related to working in tertiary care hospitals, regardless of years of experience. Similarly, a study conducted in Saudi revealed that 60% of the dentists had poor knowledge regarding BRONJ with weak positive correlation with work experience.^16^ Comparably, in a similar study conducted in Saudi, only 61% dentists were aware of the term osteonecrosis of jaw and 35% of the candidates were able to diagnose it correctly. Whereas less than 60% knew about radiations causing the disease.^17^

In our study, 43.5% of the dentists were aware of surgical treatment guidelines with knowledge related to more experience with bisphosphonates and. 41.5% of the dentists knew about endodontic management of such patients that was statistically related to working in tertiary care setups. In the same way, a study conducted in 2017 disclosed that 48% and 2.5% dentists from New Zealand and Malaysia followed formal guidelines for dental assessments and treatments for radiation candidates thus highlighting the lack of knowledge and inadequate awareness among the dental community.^18^-^20^

### Limitations:

There are a few limitations of the study that include sampling technique, the locality was limited to the twin cities only and there was no local study found to compare the data and results with.

## CONCLUSION

The inadequate awareness of dentists and medical practioners about the occurrence and management of osteonecrosis of jaw is alarming and raises high concerns about its prevention. Efforts should be undertaken to raise the knowledge regarding this preventable but extremely complicated disease through seminars and educational programs targeting both dentists and physicians to promote evidence based practice and enlighten the medical and dental faculty, ultimately leading to improved patient care.

### Author’ Contribution:

**MA, ZA:** Contributed to the idea, questionnaire and data collection.

**LA:** Contributed to the design, statistical analysis and drafting of the manuscript.

**VF, AK, MA, ZA:** Contributed to data collection.

All authors take equal responsibility for the accuracy and integrity of the work.
